# Methodology of the INVestigating traIning assoCiated blasT pAthology (INVICTA) study

**DOI:** 10.1186/s12874-022-01807-2

**Published:** 2022-12-13

**Authors:** Michael J. Roy, David O. Keyser, Sheilah S. Rowe, Rene S. Hernandez, Marcia Dovel, Holland Romero, Diana Lee, Matthew Menezes, Elizabeth Magee, Danielle J. Brooks, Chen Lai, Jessica Gill, Suthee Wiri, Elizabeth Metzger, J. Kent Werner, Douglas Brungart, Devon M. Kulinski, Dominic Nathan, Walter S. Carr

**Affiliations:** 1 Department of Medicine, Center for Neuroscience and Regenerative Medicine, Uniformed Services University, Bethesda, MD 20814 USA; 2 Henry M. Jackson Foundation, Rockville, MD USA; 3 National Institutes of Health, Bethesda, MD USA; 4 Applied Research Associates, Albuquerque, NM USA; 5 Walter Reed National Military Medical Center, Bethesda, MD USA; 6 Center for Military Psychiatry and Neuroscience, Walter Reed Army Institute of Research, Silver Spring, MD USA

**Keywords:** Traumatic brain injury, subconcussive injury, concussion, military blast exposure

## Abstract

**Background:**

Subconcussive blast exposure during military training has been the subject of both anecdotal concerns and reports in the medical literature, but prior studies have often been small and have used inconsistent methods.

**Methods:**

This paper presents the methodology employed in INVestigating traIning assoCiated blasT pAthology (INVICTA) to assess a wide range of aspects of brain function, including immediate and delayed recall, gait and balance, audiologic and oculomotor function, cerebral blood flow, brain electrical activity and neuroimaging and blood biomarkers.

**Results:**

A number of the methods employed in INVICTA are relatively easy to reproducibly utilize, and can be completed efficiently, while other measures require greater technical expertise, take longer to complete, or may have logistical challenges.

**Conclusions:**

This presentation of methods used to assess the impact of blast exposure on the brain is intended to facilitate greater uniformity of data collection in this setting, which would enable comparison between different types of blast exposure and environmental circumstances, as well as to facilitate meta-analyses and syntheses across studies.

## Background

Traumatic Brain Injury (TBI) has an estimated incidence of 15–22% in veterans of the recent wars in Iraq and Afghanistan. Mild TBI (mTBI), characterized by loss or alteration of consciousness [1], is responsible for the great majority of these cases, has been considered to represent the lower end of the TBI spectrum, and has received considerable attention in the medical literature. In the last decade, however, increasing concerns have been expressed regarding the significance of subconcussive head trauma, which by definition does not result in loss or alteration of consciousness, but may result in symptoms such as headache or dizziness. Concern originated in contact sports [2], particularly with the demonstration of chronic traumatic encephalopathy (CTE), a progressive debilitating brain disorder [3, 4] in athletes who had never experienced a concussion. Additionally, a dose–response relationship was demonstrated between a cumulative head impact index (incorporating years of exposure to impact sports, positions played, and published accelerometer data in former football players) and subsequent cognitive impairment, self-reported executive dysfunction, depression, apathy, and behavioral dysregulation [5]. In addition to exposures in the world of athletics, a growing body of evidence has been gathered suggesting that blast overpressure in military service members (SMs) may have acute, cumulative, and long-term pathophysiological effects that are unique and do not contribute to CTE [6–8].

Among Army personnel returning from deployment, blast has been implicated in 77% to 88% of deployment-related concussions [1, 9, 10]. Although subconcussive blast exposure has received far less attention, military SMs are uniquely susceptible to potential injury from blast waves generated by the detonation of high explosives or combustion of propellants inherent in various weapon systems, which can be transmitted throughout the body. There is also anecdotal evidence of post-concussive symptoms in SMs with repetitive subconcussive blast exposure (RSCBE). In a study of blast exposure in New Zealand breachers, reaction time and overall cognitive performance were associated with cumulative impulse, or total blast overpressure experienced, during two weeks of training [11]. Another study of SMs exposed to blast, as well as military law enforcement personnel not exposed to blast [6], identified a relationship between RSCBE and self-reported functional changes. Although several studies have attempted to examine relationships between RSCBE and changes in clinical and physiological function, they have been limited in size or scope, and have had inconsistent methodologies, resulting in overall equivocal findings [12–14]. However, the measurement of blast overpressure using wearable blast gauges has since become increasingly sophisticated and reproducible. The current generation of wearable blast sensors reliably measure blast overpressure and capture detailed recordings of pressure versus time as blast exposure events occur. Recording is triggered by an incident overpressure event that exceeds a preset overpressure threshold and ends after blast-related energy has dissipated [15].

The capability to measure neurocognitive [16–18], neurosensory [16, 19, 20], neuromotor [13] and autonomic nervous system [21] function has similarly become increasingly sophisticated, sensitive, and reliable. In addition, improvements in functional and structural neuroimaging make it possible to assess the potential impact of something as subtle as RSCBE which does not necessarily show up with conventional magnetic resonance imaging [22–26]. In fact, computerized axial tomography (CT) and magnetic resonance imaging (MRI) are by definition normal in cases of concussion or mild TBI, and subconcussive blast exposure would similarly not be expected to show alterations on these imaging studies. However, diffusion tensor imaging (DTI) and/or functional MRI (fMRI) [25, 26] may be more sensitive to the impact of blast. This has in fact been demonstrated in subconcussive head injuries in athletes, with DTI demonstrating an increase in white matter diffusivity [27], and fMRI showing alterations in the functional connectivity of the default mode network (DMN) [28]. There is also evidence of that fMRI changes correlate with impairment in verbal and visual memory [29]. In military SMs with blast-related mTBI [30] and blast exposure [31], DTI demonstrated reduced fractional anisotropy, consistent with white matter impairment. One form of DTI, diffusion weighted imaging (DWI), has also identified decreased fractional anisotropy and increased radial diffusivity, evidence of white matter damage, in career breachers who are repeatedly exposed to blast [32]. Resting state fMRI demonstrated increased DMN connectivity in career breachers compared to controls [32]. In summary, DTI and fMRI appear to have promise in documenting the impact of RSCBE, but the studies to date have been relatively small, with varying methodologies, and more study is needed.

In addition to alterations in neurophysiology and imaging, changes in blood levels of molecular markers may represent an especially important and easily accessible window that cannot only facilitate the identification of injury, but also may elucidate specific pathophysiological mechanisms. Notably, neuron-specific enolase (NSE) and ubiquitin carboxyl-terminal hydrolase isozyme L1 (UCH-L1) indicate neuronal cell body injury, while microtubule-associated proteins 2 (MAP2) have been associated with dendritic injury, and neurofilament-light (NF-L) and heavy chains (NF-H), tau and spectrin breakdown products have been linked to axonal injury. Elevations in glial fibrillary acidic protein (GFAP) and S100β markers represent potential blood markers of astroglial injury, whereas myelin basic protein (MBP) is more specific for myelin/oligodendrocyte damage [33–35]. S100β is also considered a marker of blood brain barrier (BBB) impairment [36–38]. A relationship has been demonstrated in athletes with repetitive subconcussive head injury (RSCHI) and some of these biomarkers, including NSE [39–41], UCH-L1 [42–46], S100β [37, 39–41, 47, 48] and tau [49]. MicroRNA analyses represent additional potential circulating markers of TBI [35, 50–54]. Among military SMs with blast exposure, there is evidence of elevations in blood levels of UCH-L1 [14], GFAP, and Spectrin Breakdown Product [SBDP]-150 [11]. Thus, blood biomarkers have particular promise in enhancing our understanding of the dimensions of impact of subconcussive blast exposure on the brain.

In summary, while there is growing evidence of the impact of repeated concussive and subconcussive head impacts in athletes, to date there has been comparatively little attention on the impact associated with military training exercises and wartime activities. The INVestigating traIning assoCiated blasT pAthology (INVICTA) study is designed to address identified gaps in knowledge regarding the impact of RSCBE incurred during heavy weapons training on the brain, through the measurement of biomarkers, as well as a series of neurocognitive, neurobehavioral, neurosensory, neuromotor, and autonomic nervous system assessments. Moreover, investigation of these gaps in the understanding of the impact of RSCBE was mandated by the U.S. Congress in Sect. 734 of the National Defense Authorization Act (NDAA) for Fiscal Year 2018 as well as in Sect. 742 of NDAA 2020. INVICTA seeks to more explicitly define a wide range of blast-related brain pathobiology, thresholds of injury, the duration of any impairments identified, and the demographic and environmental factors that may impact the injury pattern. The ultimate goal is to provide guidance to military leadership and medical personnel to mitigate risk, as well as to lead to the development of preventive or protective measures, and potential therapeutic solutions.

The purpose of this paper is to describe the unique, technologically sophisticated set of assessments employed in the INVICTA study to serially delineate a broad spectrum of brain activity and the full range of potential impact resulting from RSCBE during Special Operations Heavy Weapons Training (HWT).

## Methods

### Study hypotheses and aims

The primary hypothesis of the INVICTA study is that exposure to repeated subconcussive blast events during training with shoulder-fired heavy weapons (e.g., recoilless rifles, shoulder-mounted assault weapons, and anti-tank rockets), as quantified through the use of personally worn blast sensors, will be associated with acute and longer-term changes in the serum levels of UCH-L1, a marker of neuronal injury. The secondary hypothesis is that exposure to RSCBE during training with shoulder-fired heavy weapons, as quantified through the use of personally worn blast sensors, will be associated with changes in recall memory function. Exploratory aims are to, 1) explore the relationship between extent of blast exposure and clinical [i.e., neurocognitive, neurobehavioral, neuromotor (including oculomotor assessments), neurosensory, autonomic, vision, and hearing function] and pathophysiological (i.e., markers of neuronal, glial, vascular and blood–brain barrier damage, inflammatory and immune system reaction) outcome domains, 2) evaluate changes in acute responses to blast exposure of range safety officers (RSOs)/instructors at the start vs. end of their 2-year tour of duty, 3) explore potential subject-specific effect modifiers (factors that may alter subject response to blast exposure), 4) explore the efficacy of alternative measures of blast exposure (e.g., maximum overpressure, positive phase duration, and frequency of exposure) and related factors (e.g. type of shoulder-mounted weapon and ammunition) to predict and/or modify outcome, and 5) assess the impact of repetitive blast exposure on neuroimaging parameters (e.g., number of white matter hyper intensities, resting state functional connectivity, fractional anisotropy), by imaging RSOs both at baseline and 18–21 months later.

### Study design

INVICTA is an observational, prospective, longitudinal study featuring both within- and between-subject comparisons to better define the magnitude of RSCBE and to document the consequent cellular, neurocognitive, physiological and functional changes. Eligible participants include Special Operators and RSOs participating in HWT [i.e., firing the Carl Gustav recoilless rifle, the Light Anti-Armor Weapon (LAAW), and the AT-4 anti-tank rocket], or who are going through the same training but fire training rounds or other weapons that are found not to result in significant pressure recordings on blast gauges (training controls), and conventional active duty SMs who are not going through training and do not have blast exposure (naïve controls). The independent variable is RSCBE, measured by blast gauges mounted on helmets, shoulders and chests of participants. Outcome assessments were taken at baseline (prior to firing for exposed participants) and at 30 min, 6, 24 and 72 h, and 2 weeks (acute response), 3 months (subacute) post-firing—or associated time points for controls—for all participants, and 9 and 18 months (chronic, accompanied by a repeat of the acute and subacute response assessments (RAR) over the ensuing 3 months after the 18-month time point) for RSOs. See Table 1. This study design allows for outcome assessments to be compared with baseline for within-subject comparisons, identifying changes in assessments between the pre-exposure baseline and the post-exposure time points, and for between-subject measures between the exposed and the two control groups. It is important to note that, while Special Operators participate in HWT at 2-year intervals, RSOs typically have a 2-year tour of duty in which they conduct 6 training courses per year and are repeatedly in close vicinity to the weapons with those whose training they are supervising, which is the purpose of the longer study period and RAR assessments for that group alone.

**Table 1:**
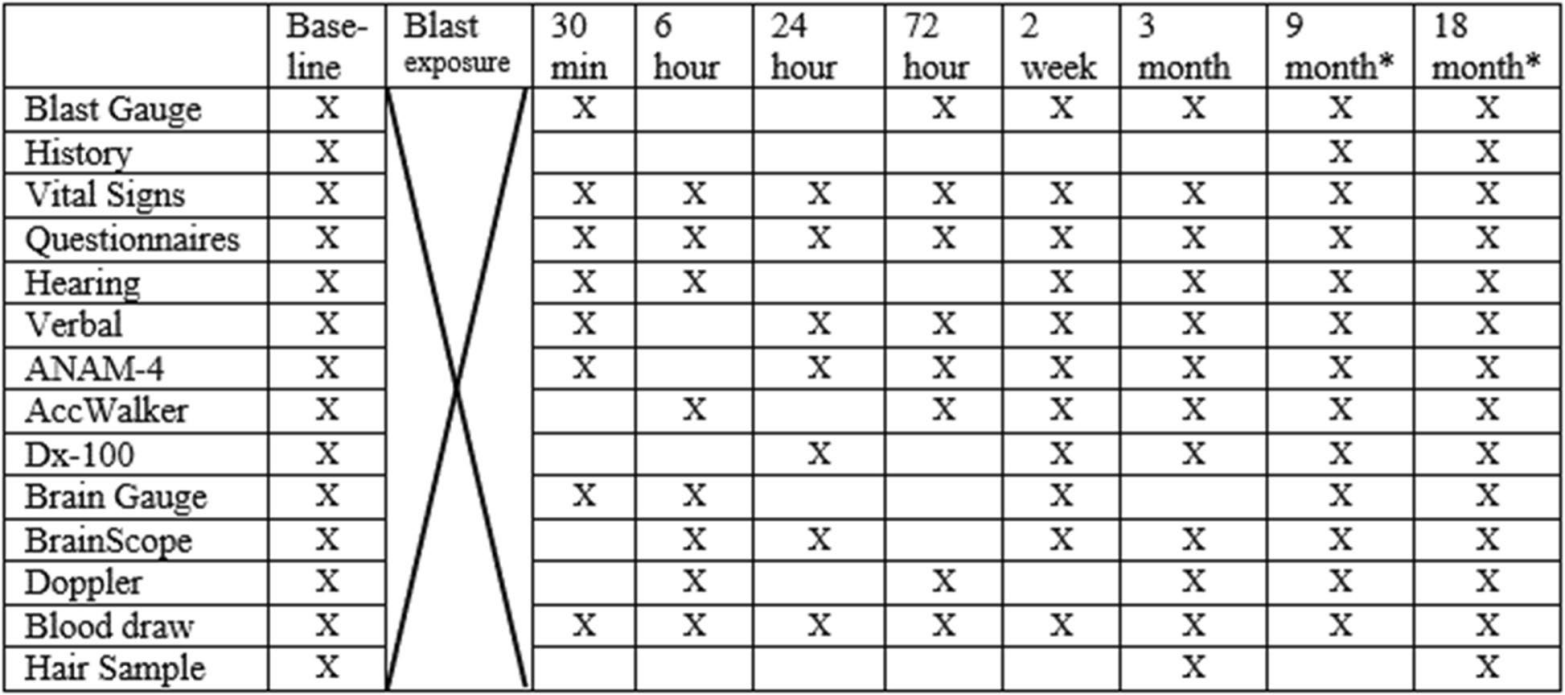
Schedule of assessments

Exposed Special Operators, Training Controls and Naïve Controls are tested Baseline through 3 months

^*^Range Safety Officers (RSOs) are followed for 21 months: Baseline through 18 months, and at 18 months the 30-min, 6-, 24-, 72-h, 2-week and 3-month assessments are repeated, for a total of 21 months of study participation

### Study population

INVICTA has a target population of 300 participants: 100 Special Operators who fire weapons of interest during HWT; 50 Special Operators who take part in HWT but do not fire weapons of interest and do not have peak blast overpressures of >  ~ 27.6 kiloPascals (kPa) or 4.0 pounds per square inch (psi) recorded on their blast gauge sensors; 100 RSOs, and 50 active duty military service members who are not Special Operators. The INVICTA study population of 200 blast-exposed (100 Special Operators and 100 RSOs) and 100 control subjects (50 Special Operators and 50 conventional active duty service members) is intended to provide sufficient power to differentiate a hypothesized 25% increase in serum biomarker levels comparing post-exposure measures with baseline. Based upon a standardized mean pre-blast exposure score value of 100, a post-exposure standardized score value of 125, a standard deviation of 100 scale points at each time point, and alpha = 0.05, we would have 94.2% power to detect this hypothesized 25% increase in serum UCH-L1 after RSCBE. Similarly, we will be able to detect a between group difference of 25% in serum levels of UCH-L1 comparing 200 exposed individuals with 100 controls with 82.3% power (alpha = 0.05), assuming a scaled score of 125 points (S.D. 100) for exposed, and a scaled score of 100 points (S.D. 50) for controls. Prior to the conduct of any assessments, informed consent is obtained from each participant by either the principal investigator or other study staff who have proven their ability to administer consent to the satisfaction of the principal investigator. The consent process includes providing the participant with a consent form that conveys detailed information about any benefits and risks, the voluntary nature of participation, and the ability to withdraw from participation at any time. This information is also conveyed verbally, time for participants to ask questions is provided, and participants are queried to confirm their understanding of key points. Both the participant and the administrator of the consent sign the form, and a copy of the signed consent is provided to each participant.

#### Assessments

### Self-reported military and health status

Participants complete each of the following:

1. Demographic questionnaire (e.g., marital status, age, race, ethnicity, education level, military history);
2. Basic medical history—including caffeine, alcohol, tobacco, medications, and supplement use. Administered at baseline, updated as needed at subsequent assessments.
3. The Ohio State University TBI Identification Method (OSU TBI-ID), a reliable and valid structured interview that documents lifetime TBI history, administered at baseline by a trained research team member, and updated as needed at subsequent assessments.

The following questionnaires are administered at baseline and 3 months for all participants, and at 9, 18 and 21 months for RSOs.

1. Patient Health Questionnaire-9 (PHQ-9) measure of depression symptom severity.
2. PTSD Checklist for DSM-5 (PCL-5) measure of PTSD symptom severity.
3. General Anxiety Disorder (GAD-7) measure of anxiety symptom severity.
4. Neurobehavioral Symptoms Inventory (NSI) measure of post concussive syndrome symptom severity.
5. Veterans RAND 12-Item Health Survey (VR-12) measure of overall functional status and quality of life.
6. Headache Impact Text (HIT-6) assessment of the impact of headaches on the ability to function at school, work, home, and in social situations.
7. Pittsburgh Sleep Quality Index (PSQI) measure of sleep quality, sleep disturbances, and sleep-related functioning.

Pain is assessed using the Defense Veterans Pain Rating Scale (DVPRS) at every time point, including the DVPRS Supplemental Questions at all time points except 30 min and 6 h collections after blast exposure.

#### Blast overpressure

The Black Box Biometrics (B3) Generation 7 (Gen 7) Blast Gauge System (BGS) is utilized to measure blast overpressure. The BGS is a collection of three devices (mounted on the back of the helmet, on the non-dominant shoulder and chest of the uniform or body armor); each device contains a pressure measurement sensor, an accelerometer, and a microprocessor. The gauges have a length, width, and height of approximately 49 mm by 37 mm by 24 mm. The gauges can be set to record pressures that exceed a specified threshold; in this study, the gauges are set to record a full wave form for all blast pressures that exceed ~ 6.9 kPa, or 1.0 psi. The microprocessor is programmed to identify potential blast-related events based upon changes in pressure measurements and, when such an event is detected, the gauge triggers and records 20 ms of pressure data.. Each gauge trigger is saved to memory along with a date and timestamp. The data from each gauge (head, chest, and shoulder) are downloaded and processed. Each waveform is baseline shifted to ensure the overpressure is zero prior to shock arrival. This is accomplished by taking the mean of the first 0.5 ms of the waveform and then shifting the entire waveform (up or down) by that value. For Gen 7 B3 gauges, the baseline shift is typically small (less than 0.68 kPa or 0.1 psi). The next step is removal of false positives from the data. Occasionally, the BGS records data that are not from a blast event. Each waveform is reviewed by a person experienced in blast physics and reviewing BGS data. False positives can take the form of sine waves, plateaus, square waves, or other non-physical waveform shapes. Flagged waveforms are removed. Then individual gauge triggers from each gauge are grouped together into a blast event. A blast event could cause one, two, or all three gauges to trigger. The magnitude of the blast as well as the body position (standing, kneeling, etc.) and orientation (facing or facing away from the blast) of the subject can influence which gauges trigger during a blast event. The date and timestamp as well as the pressure data are used in this grouping process. The BGS data represented the overpressure at the gauge location on the body. The effects of shock reflection, diffraction, or shielding can all influence the recorded value. An updated version of the FAST algorithm [54] called FAST-CT is used to estimate the incident (side-on) peak overpressure and peak overpressure impulse (a measure of energy) for each blast event. The incident blast metrics are independent of the subject body position or orientation relative to the blast.. Study participants are issued gauges at the time of baseline assessment. Participants are shown how to place the gauges on their helmet, chest, and non-firing shoulder; how to “awaken” the gauges by moving them; and how to check for cumulative overpressure exposure by pressing the recessed activation button. They are instructed to wear the gauges when participating in training events in which there is a potential for exposure to blast overpressure. Overpressure and acceleration data are downloaded from the gauges by a USB cable connected to a laptop computer at all data collection time points after the index training exposure. The data are then transferred to the Center for Neuroscience and Regenerative Medicine (CNRM) CASA secure database, where the data can be accessed for post-processing and analyses. Study staff observe and record the type of weapon fired, and the position and distance that the participant is from the weapon in a Manual Firing Line log, which also incorporates input from the study participant that is obtained by querying them immediately after the weapon firing.

#### Blood biomarkers

Blood is collected at every time point by study personnel who are trained phlebotomists following Standard Operating Procedures (SOPs). The venipuncture procedure is standard, consisting of the participant’s blood collection into two 10.0 mL Serum Separating tubes (SST) and a single 8.5 mL PAXgene DNA tube. The whole blood in PAXgene DNA tube requires no further processing beyond a room temperature incubation period for a minimum of two to maximum of 48 h. Post incubation, the PAXgene tube is then inventoried and flash-frozen as appropriate. The SSTs are processed by activating the clotting agent and allowing 30 – 45 min for incubation at room temperature. Post incubation, SSTs are centrifuged for 10 min at 1100–1300 rcf. The participant’s serum is aliquoted in 400 µl quantities into identification specific cryogenic vials. These vials are then inventoried and stored in 81 grid cryoboxes and flash-frozen as appropriate. PAXgene tubes and serum samples are processed, flash-frozen as appropriate, and shipped to the CNRM Biorepository in Bethesda, MD. Individual identified samples will be logged in, catalogued using the specimen management system of the CNRM, and maintained in -80 degrees Celsius freezers. INVICTA serum samples are analyzed in duplicate using the ultra-sensitive Single Molecule Array (SIMOA) Assay (Quanterix, Lexington, MA) for measurement of tau, neurofilament light chain (NfL), glial fibrillary acidic protein (GFAP), and Ubiquitin C-Terminal Hydrolase L1 (UCHL-1) (Human Neurology 4-Plex A assay (N4PA), Cat# 102,153) concentration on an HD-X Simoa instrument according to instructions from the manufacturer (Quanterix, Billerica, MA). Serum samples are measured in SIMOA HD-X within machine fourfold dilution. Briefly, four distinct, dye-encoded bead populations are presented with analyte-specific capture antibodies that are first incubated with samples and biotinylated detector antibodies. The target molecule present within each sample are captured by capture beads and labeled with the corresponding detector antibodies. The bead-conjugated immunocomplex is thoroughly washed and labeled with streptavidin-conjugate b-galactosidase. Following a final wash, resorufin b-D-galactopyranoside is added. The bead-conjugated immunocomplexes are loaded on the SIMOA array disc, which is designed to enable imaging of each bead via their encoded dyes and fluorescent substrate generated signals. The number of bead-containing wells producing positive signals is proportional to the number of target molecules within the sample for each plex. The average number of enzymes per bead (AEB) of each sample fit into a four-parameter logistic curve plotted using the known concentration of the calibrators. The correlation is confirmed for the accuracy of fit and for the conversion of AEB values to concentrations. We run each assay on each sample twice and determine a coefficient of variation (CV) between the two tests; we then exclude those samples from analysis if the CV exceeds 30%. The primary biomarkers of interest are ubiquitin carboxyl-terminal hydrolase isozyme L1, neurofilament-light, tau and glial fibrillary acidic protein.

#### Neurocognitive

The primary method for the assessment of neurocognitive performance is with the Hopkins Verbal Learning Test – Revised (HVLT-R). This assessment is a list learning test that consists of 12 nouns within three semantic groups using six alternate forms that evaluates verbal learning and memory. The administrator reads 12 words aloud, with an interstimulus interval of two seconds, and the participants are asked to immediately recall them. The list is read a second time followed by a second immediate recall. The list is then read a third time followed by a third immediate recall. The words recorded for each recall trial are then tallied to calculate the total recall score of all three trials. The participants are then informed that the examiner will ask them to remember the trial words at a later time during the visit. A delayed recall trial begins 20–25 min after the completion of the third immediate recall trial, during which time participants are engaged in other assessments; the participant is asked to recite from memory as many of the words on the list as possible. A percent retention is then calculated by dividing the total number of target words from the delayed recall trial by the highest score of the second or third immediate recall trial, multiplied by 100. Following the delayed recall trial is a yes/no delayed recognition trial which consists of 24 words (12 true-positive words from the original list, interspersed with 6 semantically related false-positive words and 6 semantically unrelated false-positive words). The examiner reads each word and asks the participant to respond with ‘yes’ if they believe the word was on the original list or ‘no’ if they believe it was not. The Recognition Discrimination Index is scored by calculating the total number of true-positive words minus the total number of semantically related and semantically unrelated false-positive words. The HVLT-R is administered at baseline, 30 min, 24 h, 72 h, 2 weeks, and 3 months for all participants, as well as 9 and18 months, plus a full RAR assessment series, for RSOs.

In addition to the HVLT-R, *The Automated Neuropsychological Assessment Metrics Version 4 (ANAM-4)*, a validated, widely used, computer-based tool designed to detect speed and accuracy of attention, memory, and thinking ability, is also utilized. The ANAM can assess processing speed, attention span, memory, and other cognitive components. The full ANAM Concussion Protocol battery is administered at baseline, 72 h, 18 months, and the 18 month RAR 72 h time point. At every other time point except for the 6 h and 18 RAR 6 h, at which the ANAM will not be administered, only the ANAM Reaction Time and Matching to Sample tests will be administered.

### Audiologic assessment

Auditory function, which is frequently a concern related to blast exposure, is assessed with a tablet-based hearing assessment known as TabSINT, an open source platform for administering tablet-based hearing related tests as well as questionnaires. Each tablet is paired with the Wireless Automated Hearing Test System (WAHTS) from Creare LLC. The easily portable WAHTS provides the capabilities of a fully functional audiometer and connects to the Android tablet running TabSINT via a Bluetooth connection. This system enables calibrated threshold-level audiometric measurements via the TABSINT platform. The highly attenuating earcups (or headphones) of the system provide noise isolation comparable to a single-wall booth [20]. The INVICTA audiologic assessment includes detection and discrimination tasks. For example, the participant is asked to use a button to detect the presence of a tone with and without noise presented over headphones (the Bekesy Threshold Measurements, Audiometry Fixed-Level Frequency Thresholds, and Masking Level Difference), or the participant hears a speech signal and is asked to use response buttons to identify which sound is heard (i.e., Triple Digit Test). Self-report hearing surveys are also administered to assess hearing complaints reported before and after blast, including The Hearing Screening tool (THS) and the Acute Auditory Change Questionnaire (AACQ). The THS is a validated assessment that measures the impact of tinnitus, hearing loss, and sound tolerance on a 0 (No, not a problem) to 4 (Yes, a very big problem) point scale [20]. The AACQ is used to assess hearing ability before and after blast or noise exposure [20].

A more detailed battery is delivered at baseline and 18 months for RSOs, which includes the Full AACQ, Bekesy Threshold Measurements for frequencies from 0.5-8 k Hz, Audiometry Fixed-Level Frequency Thresholds, Triple Digit Test, and Binaural Masking Level Difference Test. A shortened battery, consisting of 2 AACQ questions per ear, Bekesy Threshold Measurements for 4 and 6 k Hz only, the Triple Digit Test, and the Binaural Masking Level Difference Test is administered 30 min, 6 h, 2 weeks and 3 months after blast exposure, as well as at 9, 18 and 21 months for RSOs.

### Neuroimaging

INVICTA conducts standard structural MRI, resting state functional MRI and DTI, in RSOs at baseline when they begin their 2-year tour as a Land Warfare Training RSO, and again 18–21 months later, near the end of their tour. RSOs typically are exposed to blast over the course of 10–12 Heavy Weapons Training exercises during the intervening months. Participants are screened by study staff prior to the scan to ensure that they do not have shrapnel or other ferro-magnetic metal in their bodies that could pose a risk in the scanner. The MRI protocol employs commercially available and widely published pulse sequences, including DTI, DWI, gradient recalled echo (GRE), T1 MPRAGE, T2-weighted fluid attenuated inversion recovery (T2-FLAIR) and resting state fMRI.

### Neuromotor

**Fig. 1:**
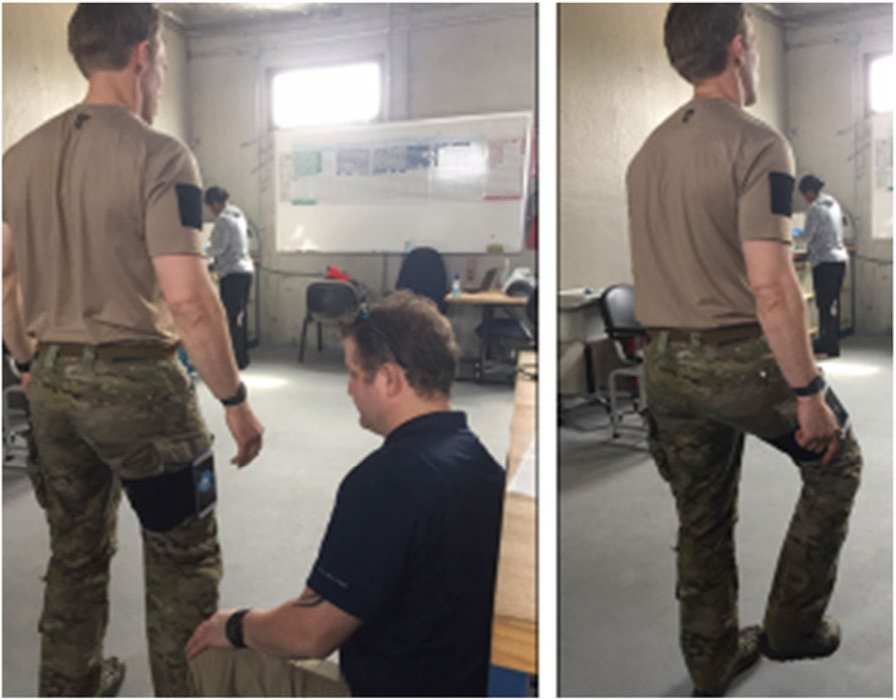
AccWalker Assessment. Legend: AccWalker assessment of balance and gait, conducted with a custom application incorporated in a cellular phone strapped to the right thigh of each study participant

Neuromotor performance is assessed with the AccWalker (Fig. 1), a valid and reliable [55] smartphone-based dynamic balance test that utilizes the phone’s to objectively measure neuromotor performance. This neuromotor assessment uses a stepping-in-place task as a dynamic challenge to the balance control system [56]. A smartphone is placed on the participant’s thigh and the AccWalker measures the thigh’s motion throughout the task. Participants are asked to step in place with eyes closed for one 30-s practice trial and two 70-s trials. Several variables are derived from the thigh motion time series that characterize temporal and spatial aspects of the movement (e.g., time between strides, range of motion of the thigh, and variability in motion between strides). The AccWalker is administered at baseline and 6 and 72 h, 2 weeks and 3 months for all participants, as well as at 9, 18 and 21 months for RSOs.

**Fig. 2:**
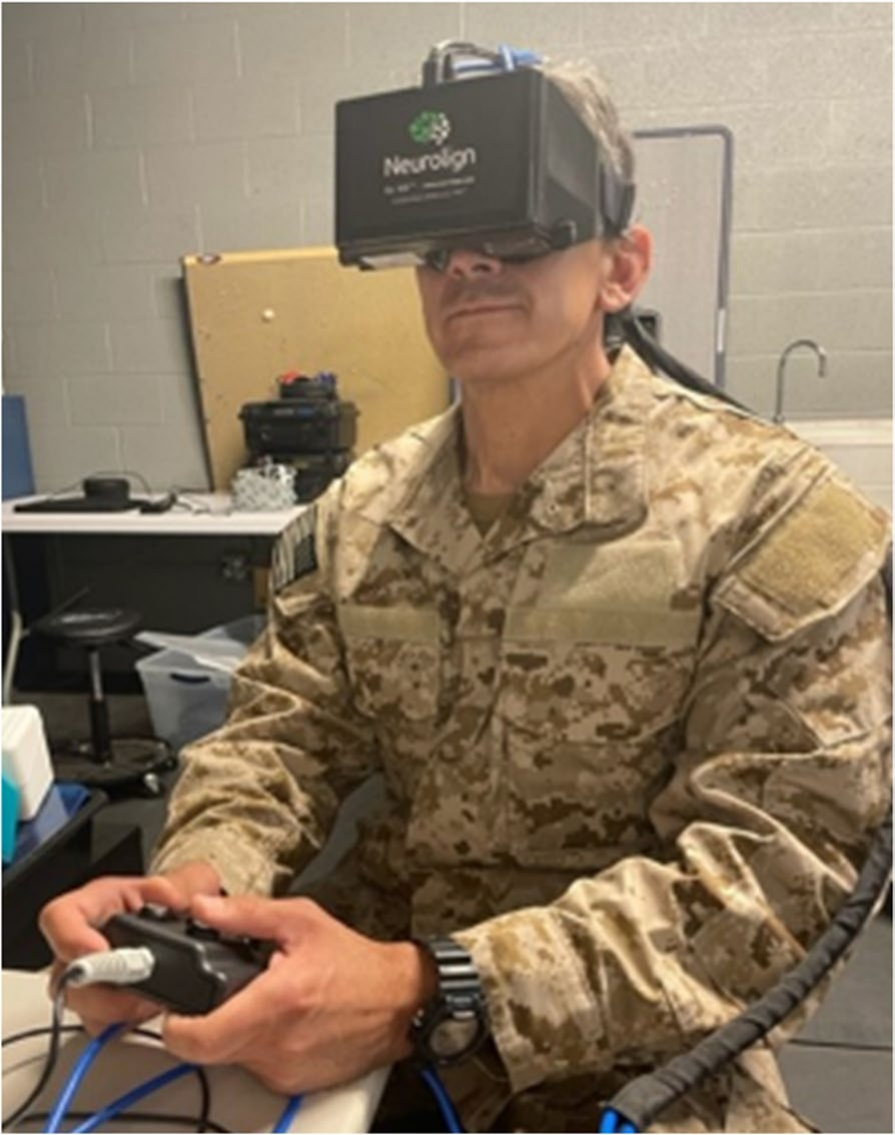
Eye Tracking Assessment. Legend: A portable head-mounted 3D display system with integrated eye tracking technology measures oculomotor and vestibular function, reaction time and cognitive performance

Oculomotor, vestibular, reaction time, and cognitive (OVRT-C) performance is measured with the Dx-100, a portable, 3D head-mounted display system with integrated eye tracking technology (Fig. 2). This FDA-cleared device provides visual and auditory stimuli and records the horizontal, vertical, and torsional movement of the eye, along with pupillary changes, and also records the participant’s visual and auditory reaction time. Participant are asked to wear the 3D head-mounted display system and complete various oculomotor, vestibular, and reaction tests following stated instructions. The Dx-100 is administered at baseline, 24 h, 2 weeks, and 3 months after blast exposure, as well as at 9, 18 and 21 months for RSOs.

### Cerebrovascular function

The Lucid™ M1 Transcranial Doppler (TCD) Ultrasound System measures cerebral vascular reserve in the Middle Cerebral Arteries (MCA) to calculate a vaso-motor reactivity (VMR) index. With participants in the supine position, a bilateral TCD monitoring exam is conducted where two transducers are simultaneously applied to the temporal region targeting the right and left MCA to record the mean velocity, pulsatility index (PI = peak systolic velocity-diastolic velocity/mean velocity), and vaso-motor reactivity index [VMR Index = 100% x (Velocity_EndC02—Velocity EndHyper/Velocity Baseline)] with breath holding and hyperventilation. This VMR monitoring exam includes a 30 s baseline recording, 30 s breath holding, 30 s recovery period, and then 30 s of hyperventilation. The TCD assessment is conducted at baseline, 6 h, 72 h, and 3 months for all participants, as well as at 9, 18 and 21 months for RSOs.

#### Neurosensory

**Fig. 3:**
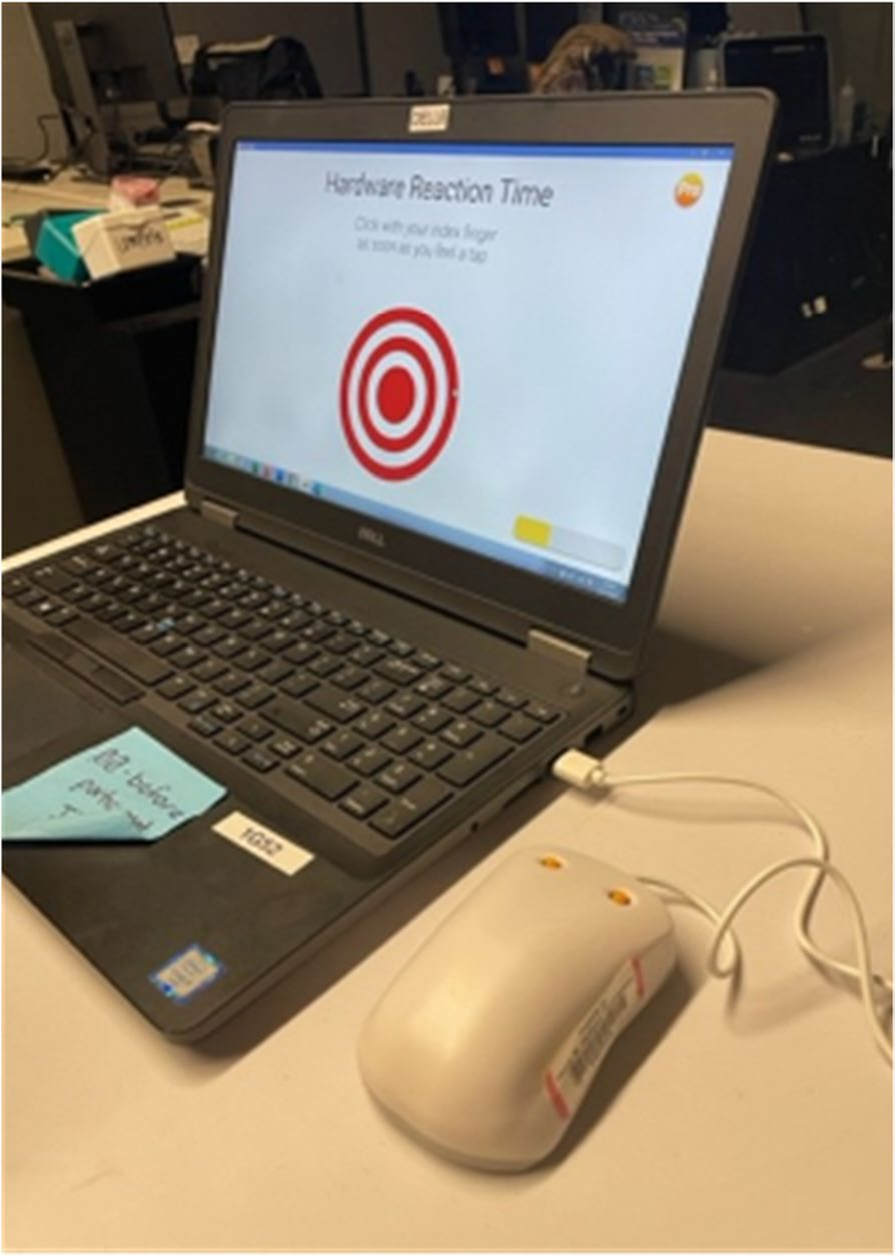
Neurosensory Assessment. Legend: Changes in tactile neurosensory functioning are examined using the custom-built two-point vertical displacement stimulator device

Possible blast exposure-related changes in tactile neurosensory functioning are examined using the custom-built two-point vertical displacement stimulator Brain Gauge™ (Corticalmetrics, LLC) device (Fig. 3). The somatosensory system has been suggested to be highly sensitive to brain injury-related changes in function. The organization of the somatosensory system enables the study of cortical–cortical interactions in adjacent or near-adjacent cortical regions using a tactile interface device that measures the variability in human reaction to tactile stimuli. Alterations in sensory perception occur in parallel with alterations in systemic cortical alterations. The Brain Gauge device utilizes gentle mechanical stimulation (vibration) via computer-generated sinusoidal waveforms that are relayed to mechanical transducers that stimulate the fingertips. The vibrations) are delivered at varying amplitudes (0–300 microns) at constant frequency (25 Hz) for short durations (less than or equal to 750 ms), via a flat Delrin probe (5–10 mm in diameter) positioned to make contact with the index and middle finger of one hand (D2 and D3). The device is low-powered and requires only a single connection via USB with the computer or laptop that is used to administer the test(s), which makes it particularly useful in the remote site trailers in which it is administered for this study. The stimulator can apply single site or two-site stimulation to digits two or three at specified amplitudes or frequencies. Tests can be conducted to assess reaction time, amplitude discriminative capacity, frequency discriminative capacity, temporal order judgment, timing perception, and effects of conditioning stimuli on discriminative capacity. Data are collected as quantitative values of neurosensory perceptual metrics and are automatically stored. A full Brain Gauge Assessment is administered at baseline and 2 weeks post shoot; partial assessment is completed at all other time points.

Elements of the Brain Gauge test battery include the following:

- *Reaction Time* (RT) [1, 19, 57–63] Participant receives a single pulse to the middle finger; participant clicks the mouse button as soon as they feel the pulse. There is no on-screen button to click, clicking anywhere on the screen will record their reaction time and progress the trial.
- *Amplitude Discrimination* (AD) [19, 57–67] Participant compares two vibrations. Vibrations will be delivered sequentially or simultaneously to the left (index) and right (middle) fingers. Participant is directed to pick the larger / stronger / more intense vibration by clicking on the left or right button on the screen. Response time is not important for this task, allowing the participant to make the best choice possible.
- *Temporal Order Judgement* (TOJ) [19, 58, 61, 67–70]. Participant receives two pulses – one to each finger—with one preceding the other. The participant is asked to indicate “Which one came first?” The participant responds using the USB mouse to indicate LEFT or RIGHT on the screen of the computer. The participant should pick the “first stimuli” by selecting the right or left button on the screen of the computer.
- *Duration Discrimination* (DD) [19, 58, 61, 63, 64, 67]. Participant receives two vibrations of different durations. One vibration is delivered to one digit, and after a brief pause the other is delivered to the adjacent digit. Participant indicates which stimuli was longer.

#### Neurophysiology

Electroencephalography (EEG) is obtained through the use of the BrainScope One™ a handheld brain injury assessment device that uses digital EEG technology. Raw patterns of EEG signal are recorded by the device and the manufacturer’s proprietary brain function index values provide an EEG-based Brain Function Index that compares the participant’s brain function to an age-regressed normed population, expressed as a percentile (0–100). Quantitative EEG testing is performed at baseline, 6 h, 24 h, 2 weeks and 3 months to all participants. In addition, it will be administered to RSOs at 9 months and at the 18 RAR at 6 h, 24 h, 2 weeks and 3 months.

#### Statistical analyses

The ultimate goal of the INVICTA study is to develop and validate a risk stratification system, which we intend to call RSCBE Assessment Tool to Identify No-Go/Go (RATING), which will incorporate the most effective of the above measures for distinguishing the level of risk each individual would have in the event of subsequent subconcussive blast exposure.

We will apply sophisticated machine learning methods to identify and weight the most powerful study measures in order to develop and validate a RATING risk stratification system, enabling individual prognostic information to incorporate into military command decision-making and facilitate prospective protection.

Development of RATING will be based on data from a wide range of acute and subacute time points after initial firing (independent variables) and their association with two key dependent measures, the HVLT-R immediate recall measure, and the AccWalker measure of gait and balance. Validation will be performed with the same independent and dependent variables from the 18-mo Repeated Acute Response (RAR) data obtained from RSOs who repeat all the same assessments from 30 min to 3 months after another HWT RSCBE.

Machine learning techniques, including Support Vector Machines (SVM) and Neural Networks (NN), will also be essential to this analysis. We will limit Type I and Type II error by specifically identifying each analysis as Hypothesis Testing vs Hypothesis Generating. To reduce the likelihood error arising from multiple comparisons, Bonferroni corrections and other appropriate techniques will be applied. Where analyses are based upon prior knowledge, Bayesian statistical methods will be employed and the Bayes Factor will assess significance.

RATING, based on the results of assessments conducted through the first 3 months after HWT, will classify Operator performance on a 1–14 point scale. The confidence of the at-risk/not-at-risk assessment can be reinforced through a variety of statistical learning algorithms. More than one algorithm will be used, and the results obtained from the various methods will be compared, contrasted, and ultimately combined. This is accomplished by iterative processes of analysis whereby individual datasets are taken out to measure the impact on outcome analysis (i.e. weigh variables) and utilization of bootstrapping techniques to identify significant time points and datasets.

Identification of the pre-exposure, independent variables with the most power to predict exposure outcome group is critical to the development of the optimal RATING instrument. The intent is to translate this predictive power into a 14-point RATING scale to characterize the quantitative degree of risk associated with additional blast exposure as an element of performance readiness, to thereby inform go/no-go decision making as well as enable return-to-duty decisions. For deployment, command may determine that a score as high as 10 or 11 is permissible, while for a training exercise, a score of 7 or more may be sufficient to either deter from firing a particular weapon or set of weapons, or to implement additional safety measures to better protect a Special Operator.

## Discussion

Blast exposure during military training may have a significant impact on the brain health of military SMs. However, the published literature to date has been limited with regard to the number of participants and scope of assessments; moreover, study methods have been varied, making it hard to combine data across studies. The comprehensive, wide-ranging assessments employed in the INVICTA study significantly expand both the level of detail and the breadth of functional assessments of brain function in the acute, subacute and chronic phases after training-associated blast exposure. The explicit documentation of the methods being utilized may provide significant direction to others planning similar evaluations, in order to facilitate greater standardization of methods. This will enable future comparison of the impact of blast in a variety of different settings, such as breacher exercises or bomb disposal, in addition to HWT. Utilization of comparable measures across studies will also afford greater power to discern the impact of blast by facilitating the merging of data and the conduct of meta-analysis.

To further facilitate future research, it is important to recognize that some assessments are far more facile to conduct than others. The blood draw, HVLT-R, ANAM, Brain Gauge, and AccWalker are all relatively efficient and easy to perform. On the other hand, several of the measures may present logistical challenges. The TCD requires space to be able to have the participant lie supine, and requires holding probes in place for a relatively lengthy time period in order to record through the breath-holding and hyperventilation exercises. The BrainScope may require thorough, sometimes unpleasant abrading of the skin surface in order to facilitate optimal impedance measures. In addition, to maintain the conductivity of the electrode gel the headsets must be stored in a climate-controlled environment, which may be difficult to achieve in some field settings. The Dx-100 is likewise sensitive to humidity and cold temperatures, and may fog up in such conditions, though the use of simple hand-warmers and OTC anti-fog spray can effectively combat this issue.

Finally, in addition to issues with specific assessments, there are some challenges that may be encountered with field settings overall. For example, we utilized custom-made trailers with uniquely structured rooms to facilitate the various assessments and mobility to the training site. We have had logistical difficulties with the hydraulic systems that raise, lower and level the trailers, a requirement when staging for research in austere military training sites. In addition, we have had some difficulties maintaining proper functioning of the heating, ventilation and air conditioning systems, and having maintenance personnel readily available to help with such issues may be important in order to facilitate the efficient and complete collection of study data.

## Conclusions

It is hoped that this description of the INVICTA study methods, and the challenges encountered, will better enable other researchers to more efficiently set up and conduct future studies.

## Data Availability

Not applicable, as this paper describes study methodology and does not provide data.

## Abbreviations

AACQ: Acute Auditory Change Questionnaire
ANAM-4: Automated Neuropsychological Assessment Metrics
ARA: Applied Research Associates
AT-4: Anti-Tank weapon (84 mm, shoulder-launched, single shot recoilless weapon)
B3: BlackBox Biometrics
BBB: Blood Brain Barrier
BGS: Blast Gauge System
CASA: Collections Access Sharing and Analytics platform (CNRM Data Repository)
CNRM: Center for Neuroscience and Regenerative Medicine
CT: Computerized axial tomography
CTE: Chronic Traumatic Encephalopathy
DMN: Default Mode Network
DTI: Diffusion Tensor Imaging
DVPRS: Defense Veterans Pain Rating Scale
DWI: Diffusion Weighted Imaging
Dx-100: Neurolign Dx-100™, eye tracking technology
EEG: Electroencephalography
fMRI: Functional Magnetic Resonance Imaging
GAD-7: General Anxiety Disorder
GFAP: Glial fibrillary acidic protein
HIT-6: Headache Impact Test
HVLT-R: Hopkins Verbal Learning Test—Revised
HWT: Heavy Weapons Training
INVICTA: INVestigating traIning assoCiated blasT pAthology
kPa: KiloPascal
LAAW: Light Anti-Armor Weapon
MBP: Myelin Basic Protein
MCA: Middle cerebral arteries
MRI: Magnetic Resonance Imaging
ms: Millisecond
mTBI: Mild Traumatic Brain Injury
N4PA: Neurology 4-Plex A assay
NDAA: National Defense Authorization Act
Nf-L: Neurofilament-Light chain
NSE: Neuron-Specific Enolase
NSI: Neurobehavioral Symptoms Inventory
OSU TBI-ID: The Ohio State University TBI Identification method
OVRT-C: Oculomotor, Vestibular, Reaction Time and Cognitive
PCL-5: PTSD Check List for DSM-5
PHQ-9: Patient Health Questionnaire-9
PI: Pulsatility Index
psi: Pounds per Square Inch
PSQI: Pittsburgh Sleep Quality Index
RAR: Repeated Acute Response
RATING: RSCBE Assessment Test to Identify No-Go/Go status
rcf: Relative Centrifugal Force
RSCBE: Repetitive Subconcussive Blast Exposure
RSCHI: Repeated subconcussive head injury
RSOs: Range Safety Officers (which are Land Warfare Training Instructors)’
RT: Reaction Time
SBDP: Spectrin Breakdown Product
SM: Service Member
SOP: Standard Operating Procedure
SST: Serum Separating Tube
TabSINT: Tablet-based Speech-In-Noise Test
TBI: Traumatic Brain Injury
TCD: Transcranial Doppler ultrasound
THS: The Hearing Screening tool
UCH-L1: Ubiquitin Carboxyl-terminal Hydrolase isozyme L1
USU: Uniformed Services University of the Health Sciences
VMR: Vaso-Motor Reactivity
VR-12: Veterans RAND 12-Item Health Survey
WAHTS: Wireless Automated Hearing Test System
